# Clonal hematopoiesis is associated with progression of idiopathic pulmonary fibrosis

**DOI:** 10.1172/jci.insight.198458

**Published:** 2026-08-24

**Authors:** Lori Asarian, Jianlong Jia, Dmytro Sirokha, Lara Paulini, Daniela Dietel, Mircea Gabriel Stoleriu, Marion Frankenberger, Ali Önder Yildirim, Katharina S. Götze, Juergen Behr, Gary M. Hunninghake, Isis E. Fernandez

**Affiliations:** 1 Institute of Lung Health and Immunity and Comprehensive Pneumology Center with the CPC-M bioArchive, Helmholtz Center Munich, Member of the German Center for Lung Research, Munich, Germany.; 2 Department of Medicine V, LMU University Hospital, Ludwig Maximilian University (LMU) Munich, Member of the German Center for Lung Research, Munich, Germany.; 3 Division of Thoracic Surgery, LMU Munich, Munich, Germany and Asklepios Lung Clinic, Gauting, Germany.; 4 Technical University of Munich, School of Medicine, Department of Medicine III, Munich, Germany. German Cancer Consortium (DKTK), Heidelberg, Partner Site Munich, Germany. Bavarian Center for Cancer Research (BZKF), Munich, Germany.; 5 Pulmonary and Critical Care Division, Brigham and Women’s Hospital, Harvard Medical School, Boston, Massachusetts, USA.

**Keywords:** Aging, Pulmonology, Biomarkers, Fibrosis, Genetic risk factors

## Abstract

Clonal hematopoiesis-related mutations associate with rapid IPF progression and lung function decline. Exploring them mechanistically may identify important diagnostic or therapeutic targets for IPF patients.

**To the Editor:** Idiopathic pulmonary fibrosis (IPF) is chronically progressive, age associated, and fatal (1). Patients have either stable courses or progress rapidly toward death. Genetic susceptibility, telomere attrition, and epigenetic programming contribute to IPF’s etiology, but what contributes to the heterogeneous disease progression is unclear. While short telomere length is emerging as one such risk factor (2), other factors remain less understood.

Clonal hematopoiesis (CH), defined as expanded somatic blood cell clones in persons without other hematological abnormalities, is also age related (3). CH at variant allele frequencies (VAF) ≥2% is associated with greater risk of chronic diseases, such as coronary heart disease or chronic obstructive pulmonary disease (COPD) (3–5). Commonly mutated genes (e.g., *DNMT3A* and *TET2*) epigenetically control gene expression and are important regulators of disease-related immune responses (6). Given these shared risk factors (1, 3), we hypothesized that incidence of CH is associated with IPF progression.

DNA was extracted from whole blood after written informed consent was obtained from 123 patients with IPF (from the CPC-M bioArchive, Munich, Germany) diagnosed according to ATS/ERS/JRS/ALAT criteria (1), prior to lung transplantation. Clinical data, available at sampling and within 12 months after blood collection, was used to assign progression (*n* = 89) as follows: (a) ≥10% absolute forced vital capacity (FVC) 12-month decline alone; (b) ≥5% absolute FVC 12-month decline and radiologic progression; or (c) 12-month radiologic progression alone (1). CH-defining somatic mutations were analyzed with a targeted sequencing panel (*ASXL1*, *CALR*, *CBL*, *DNMT3A*, *JAK2*, *MPL*, *PPM1D*, *SF3B1*, *SRSF2*, *TET2*, *TP53*, *U2AF2*, and *ZRSR2)*. Mutation- and variant-calling analyses were done by Munich Leukemia Laboratory. Patient characteristics, sample processing methods, mutation characterization, statistical analyses, and author contributions are detailed in the Supplemental Methods (supplemental material available online with this article; https://doi.org/10.1172/jci.insight.198458DS1).

The associations of CH with demographic and clinical characteristics and disease progression are shown in Supplemental Tables 1 and 2 and Figure 1. CH mutations were present in 38% of patients with IPF (overall median VAF = 6.2%; Supplemental Table 1). Significantly more CH-mutated patients were characterized as rapid progressors (53% vs. 25%, *P* < 0.01) and had a greater 12-month loss in FVC compared with those without CH (280 vs. 90 mL, *P* < 0.01, Figure 1, A and B). Age had no significant effect (Figure 1C). CH increased odds of disease progression and associated with a decline in FVC, even when adjusting for basic characteristics, comorbidities, antifibrotics, and baseline severity (Supplemental Table 3).

Although CH and COPD have been linked (4, 5), in our cohort, only 6 patients fulfilled GOLD criteria. CH also did not influence the frequency of other comorbidities in patients with IPF, despite the known associations (Supplemental Table 4). Furthermore, although adverse outcomes after lung transplant have been associated with CH mutations (7, 8), we believe our results are unique in demonstrating an association of CH and rapid IPF progression prior to lung transplantation.

Irrespective of disease progression, multiple gene mutations (≥2 and up to 5) were present in 47% of patients, and 38% had multiple point mutations of the same gene. *DNMT3A*, *TET2*, and *PPM1D* were the most frequently mutated genes (41%, 13%, and 11%, respectively). Significantly more patients had *DNMT3A* versus other mutations (Figure 1D). Importantly, 90% of *TET2* mutations were detected in rapidly progressive patients (Figure 1E).

Missense mutations predominated, although other key protein-altering mutation types were also found (Figure 1F). Clone size was not associated with progression. In rapidly progressive patients *DNMT3A*, *TET2*, and *SF3B1* were the most frequent mutations (32%, 24%, and 16%, respectively), mostly in functional domains (74%, 55%, and 100%, respectively; Figure 1G). Variants of unknown significance were observed in 10 patients.

One critical limitation of this study is the need of replication in independent, larger populations that include similarly aged, healthy control groups. This may increase the power to detect other important effects of CH in IPF. Furthermore, given the emerging role of telomere biology in IPF and CH (2, 8, 9), future studies would benefit from adding paired telomere length measurements. Finally, the 12-month follow-up window and a small number of deaths underpowered any CH and mortality association analyses. Long-term follow-ups assessing the role of CH with IPF mortality would be of particular interest.

We believe that the significant association of CH with rapid disease progression unveils a novel mechanism underlying IPF pathogenesis, which along with identifying changes in telomere attrition and epigenetic alterations may facilitate identification of important diagnostic or therapeutic targets.

## Funding support

This work is also the result of NIH funding, in whole or in part, and is subject to the NIH Public Access Policy. Through acceptance of this federal funding, the NIH has been given a right to make the work publicly available in PubMed Central. This study was funded by the following:

- European Research Council (ERC) under the European Union’s Horizon Europe research and innovation programme (ERC StG OMEGA; grant agreement No. 101164864), to IEF.
- LMU Excellence funds to IEF.
- Helmholtz Munich institutional funds to IEF.
- NIH grants R01 HL111024, R01 HL135142, and R0130974 to GMH.

## Conflict of interest

The authors have declared that no conflict of interest exists.

## Supplementary Material

Supplemental data

Supporting data values

## Figures and Tables

**Figure 1:**
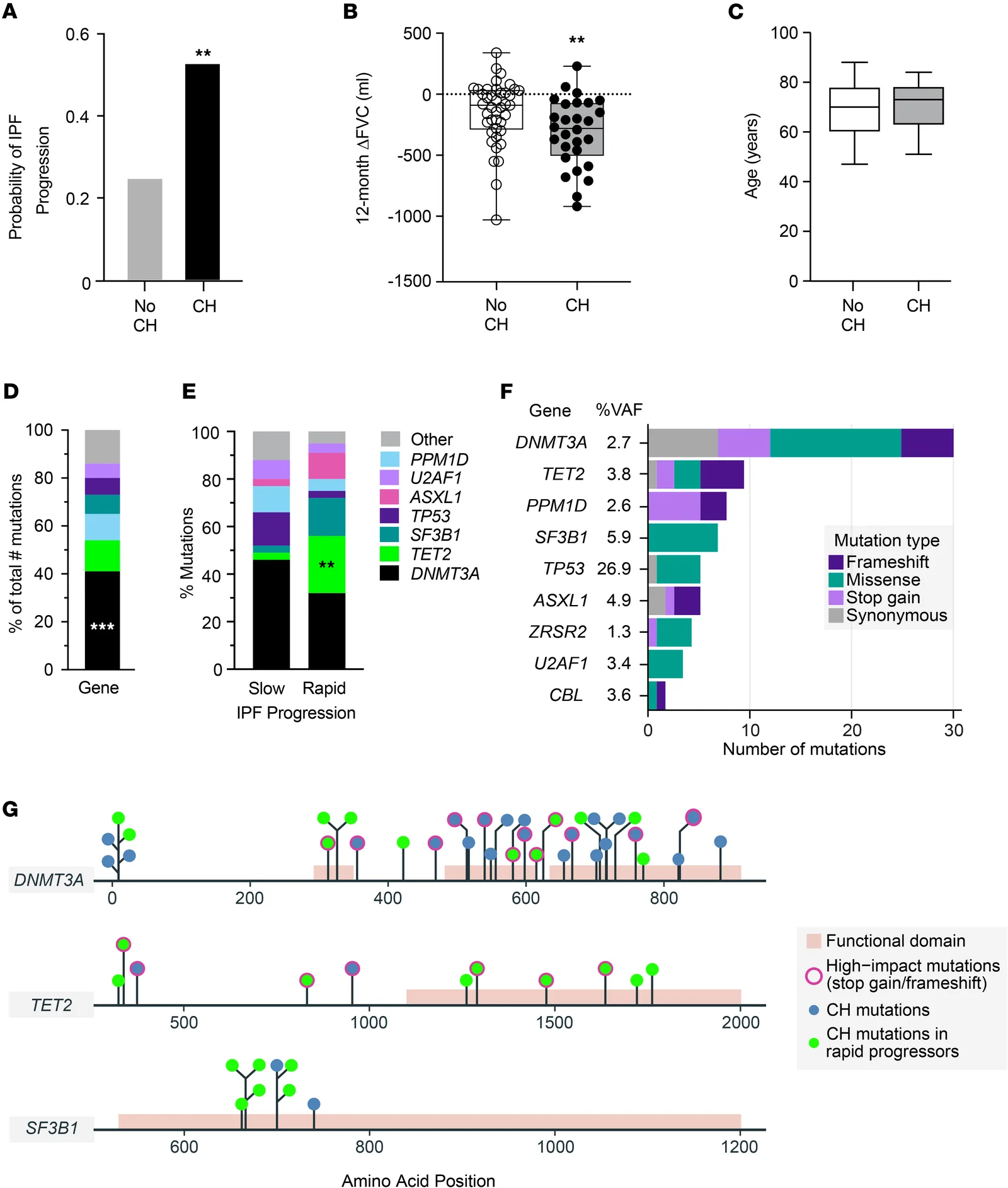
CH mutations are associated with IPF progression and loss of lung function. (**A**) Rapid IPF progression is more prevalent in patients with CH mutations (*n* = 89 patients, 51 without CH and 38 with CH; Fisher’s test, **P < 0.01, median with min/max data). (**B**) The 12-month FVC loss was significantly greater in patients with CH mutations (*n* = 41 without CH and *n* = 28 with CH, Fisher’s test; **P < 0.01, median with min/max data). (**C**) Age did not significantly affect the presence of CH mutations. (**D**) Overall, *DNMT3A* was the more prevalent mutation (binomial test, ***P < 0.001). (**E**) *TET2* mutations predominated in rapidly progressing patients (Fisher’s exact, **P < 0.01, unadjusted). (**F**) Frequencies and mutation types with representative median percentage VAF. (**G**) Protein positions of coding mutations by gene. Orange bars indicate functional domains, and mutations are shown as individual or multiple stacked events at the same position: magenta indicates high-impact mutations, blue indicates CH mutations, and green indicates rapidly progressive patients.

## Version 1 08/24/2026

Electronic publication
