# Supplementary material for: Clonal hematopoiesis is associated with progression of idiopathic pulmonary fibrosis
Source: JCI Insight. 2026 Aug 24;11(16):e198458. doi: 10.1172/jci.insight.198458 (PMC13502164; doi:10.1172/jci.insight.198458)
Supplement: Supplementary file 1 [file jciinsight-11-198458-s295.pdf]

Supplementary Methods

Sex as a biological variable

IPF is a male-predominant disease, therefore the sex of the patients in this study reflects this predominance (80%). As a result, and as is the case with human IPF studies, the statistical analyses are underpowered to determine sex differences in this study.

Study participants and ethical approval

Whole blood for gDNA extraction was obtained after written informed consent from 123 IPF patients (demographics presented in Supplementary Table 1) from the CPC-M bioArchive (CPC Munich, Germany). The study was approved by the local ethics committee of the Ludwig-Maximilians University of Munich, Germany (Ethic vote #19-629). IPF was diagnosed according with ATS/ERS/JRS/ALAT criteria (1). IPF progression was assessed using absolute decline in FVC% predicted and/or radiologic progression in patients with data available within 12 months of follow-up after blood collection using one of the following criteria: 1) thresholds of lung function decline alone (absolute FVC decline ≥10%, n=62), 2) ≥5% absolute FVC decline in combination with radiologic changes (n=7) or 3) radiological changes alone (n=20) (2,3). Radiographic progression (worsening fibrotic changes on serial chest computed tomography) was defined according to ATS and ERS established criteria (3). Data for progression assessment were available for 89 patients. COPD stages were assessed using standard GOLD staging criteria (4). Patients with FEV1/FVC ratio below 0.7 and the following predicted FEV1 (pFEV1) parameters: 1) Mild=greater or equal to 80%; 2) Moderate= 50-79%; 3) Severe=30-49%; 4) Very severe=less than 30% (4) were assigned as COPD GOLD stages 1 to 4, respectively.

Sample preparation

Pelleted whole blood was processed with DNeasy Blood and Tissue kit (Qiagen, Hilden, Germany) following manufacturer's kit instructions. Subsequently, DNA concentration was measured with a high-sensitivity DNA Qubit kit (Thermo Fisher

Scientific, MA, USA). DNA samples were then appropriately diluted and sent for targeted gene sequencing.

Targeted CH panel gene sequencing

Analysis of somatic mutations defining clonal hematopoiesis (CH) was performed with a targeted gene panel including 13 genes known to be most frequently mutated (*ASXL1, CALR, CBL, DNMT3A, JAK2, MPL, PPM1D, SF3B1, SRSF2, TET2, TP53, U2AF2*, and *ZRSR2*). The library preparation for enrichment was performed from 150ng DNA per sample with the Illumina DNA Prep Kit (Illumina, San Diego, CA, USA) using Unique Dual Indices and an enzymatic DNA fragmentation. Target enrichment was done using the IDT Hybridization Capture Protocol and a corresponding lockdown gene panel (IDT Integrated DNA Technologies, Coralville, IA, USA). Sequencing was performed on a NovaSeq 6000 (Illumina) with paired end sequencing mode (2x101 cycles) and a target coverage of 2,000x. De-duplication was performed to eliminate duplicated, identical PCR reads before alignment to improve accuracy of the calculated variant allele frequency (VAF). Variant calling of single nucleotide variants and small InDels (<50 base pairs) was carried out with the variant callers Dragen, Pisces (Illumina) and Pindel (Wellcome Sanger Institute, Cambridgeshire, UK) followed by a manual annotation and classification of the variants by in-house routine accredited (ISO 15189 and College of American Pathologists) workflow of the Munich Leukemia Laboratory (MLL, Munich, Germany). This workflow uses open access databases like COSMIC, IRAC, ClinVAR, dbSNP und gnomAD, as well as the in-house databases specialized on hematologic diseases. Protein truncating and splice site affecting variants were classified as mutation. Non-synonymous changes were included, considering different aspects: variants were well annotated for pathogenicity in hematologic malignancies (several definite submissions to COSMIC, IRAC, ClinVAR, or MLL database), within a functional domain of the protein or likely somatic based on a VAF <25%; for *ASXL1* only protein truncating variants were considered pathogenic. Other rare non-protein-truncating variants were defined as variants of uncertain significance and were not considered in further analyses. Sequencing was performed at a limit of detection of 1% VAF, and detected variants with

a VAF <5% were independently confirmed by amplicon sequencing on a MiSeq (Illumina). High-impact CH mutations were defined as those predicted to disrupt the structure and/or functionality of the resulting protein, such as frameshift and stop gain alterations (5).

Statistical analyses

Statistical analyses focused on two questions. The first was to determine the prevalence of CH mutations in IPF, regardless of disease progression. This was done using all sequenced patients available (n=123). The second question was to determine whether CH was more prevalent in rapidly progressing patients. This was done only in a subgroup of patients (n=89) for whom we had clinical data and disease progression could be assessed. The effects of CH on categorical variables that included sex, smoking history, family history of PF, disease progression, lung transplantation, antifibrotic treatment use, mortality and comorbidities such as, gastro-esophageal reflux disease (GERD), pulmonary arterial hypertension (PAH), hypertension, coronary heart disease (CHD), other cardiovascular diseases, and malignant diseases were assessed using two-tailed Fisher's exact tests. Two-tailed Wilcoxon-Mann-Whitney tests were used to determine the effects of CH on continuous variables (age, MCV, hemoglobin, %RDW and blood counts, baseline FVC, predicted FEV1%, FEV1/FVC ratio and 12-month loss in FVC). Type I errors were corrected with Bonferroni-Holm post-hoc analyses. Clinical associations of CH, disease progression and 0-to-12-month change in FVC were assessed using multivariable logistic or linear regressions, respectively, using 3 models adjusting for 1) only basic categorical covariates (sex, smoking history, family history of PF); 2) basic categorical covariates further adjusting for CHD and malignant disease as CH-related comorbidities and 3) basic categorical covariates further adjusting for baseline disease severity (0-month FVC and use of antifibrotics). The frequencies of overall and progression-specific CH mutations were tested with binomial and Fisher's exact tests, respectively. Significance level for all analyses was $p<0.05$. Analyses and graphs were done using SAS statistical package (Cary, NC, USA), R v.4.1.2 (packages dplyr, stringr, and ggplot2) and GraphPad Prism (v. 10, San Diego, CA, USA).

Data availability

Raw data used to generate Figure 1 and Supplementary Tables 1-4 can be found in the Supporting Data File.

Acknowledgements

We gratefully acknowledge the provision of human biomaterial and clinical data from the CPC-M bioArchive and its partners at the Asklepios Biobank Gauting, the LMU Hospital and the Ludwig-Maximilians-Universität München. We thank the patients and their families for their support. We also acknowledge the support of Ronan Le Gleut and Elmar Spiegel from the Core Facility Statistical Consulting at Helmholtz Munich (CF-STATCON) for help with the statistical modeling.

Declarations

AI tools were not used in the preparation of this manuscript.

Funding Support

This study was supported by funds from Helmholtz Munich, LMU Excellence funds and ERC StG OMEGA 101164864 to IEF. GMH is supported by NIH grants RO1 HL111024, RO1 HL135142, and RO130974. This work is also the result of NIH funding, in whole or in part, and is subject to the NIH Public Access Policy. Through acceptance of this federal funding, the NIH has been given a right to make the work publicly available in PubMed Central.

Supplementary references

1. Raghu G, et al. Diagnosis of Idiopathic Pulmonary Fibrosis. An Official ATS/ERS/JRS/ALAT Clinical Practice Guideline. Am J Respir Crit Care Med. 2018; 198:e44.

2. Newton CA, et al. Performance Characteristics for Physiological Measures of Progressive Pulmonary Fibrosis. Am J Respir Crit Care Med. 2025; 211:1867.

3. Raghu G, et al. Idiopathic Pulmonary Fibrosis (an Update) and Progressive Pulmonary Fibrosis in Adults: An Official ATS/ERS/JRS/ALAT Clinical Practice Guideline. Am J Respir Crit Care Med. 2022; 205:e18.

4. Celli BR, MacNee W; ATS/ERS Task Force. Standards for the diagnosis and treatment of patients with COPD: a summary of the ATS/ERS position paper. Eur Respir J. 2004; 23:932.

5. MacArthur DG, et al.A systematic survey of loss-of-function variants in human protein-coding genes. Science. 2012; 335:823.

Supplementary Table 1. Demographics, disease progression and clinical characteristics in IPF patients

Variable	No CH	CH	*p*-value
n (%)	76 (62)	47 (38)	
**Demographic characteristics**			
Age (y), median (IQI)	71 (61-78)	73 (65-81)	0.3
Male sex (%)	86	81	0.6
**Smoking history**			0.4
Never- smoker (%)	41	33	
Ex-smoker (%)	59	67	
**Family History of PF (%)**	8	5	0.7
**Disease progression (%)**			0.01
Slow	75	47	
Rapid	25	53	
**Lung Transplantation (%)** (post-sampling date)	19	15	0.8
**Patients on antifibrotic treatment (%)**	86	74	0.2
**Mortality Rate (%)**	8	0	0.1

Data are n (%) or median (interquartile intervals, IQI) of demographic characteristics (age and sex), smoking history, family history of pulmonary fibrosis (PF), disease progression, lung transplantation events, patients on antifibrotic treatment and mortality rate. Progression was assessed over 12 months following the sampling date, as described in the supplementary methods. Because 12-month clinical data were not available for some patients, disease progression was analyzed in a subset of 89 out of 123 patients (No CH=51, CH=38). Lung transplantation and mortality events occurred post-sampling date and within the 12-month follow-up period. Significances were based on Fisher exact tests ($p<0.05$), as detailed in the Supplementary methods.

Supplementary Table 2. Effect of CH on blood and pulmonary function parameters in IPF patients

A. Baseline differential blood parameters			
**Variable (median IQI)**	**No CH**	**CH**	***p*-value**
Hemoglobin (g/dL)	14 (13-15)	14 (13-15)	0.5
MCV (fL)	90 (86-93)	92 (88-94)	0.2
RDW%	14 (13-15)	14 (13-15)	0.3
Lymphocytes (g/L)	2 (1-2)	2 (1-2)	0.2
Neutrophils (g/L)	6 (5-7)	6 (5-9)	0.2
Monocytes (g/L)	0.7 (0.6-0.9)	0.7 (0.6-0.9)	0.6
Platelets (g/L)	234 (208-285)	215 (161-261)	0.1
**B. Pulmonary function test parameters**			
**Variable (median IQI)**	**No CH**	**CH**	***p*-value**
Baseline FVC % predicted	67 (55-78)	68 (51-82)	0.9
Baseline FEV1 % predicted	73 (58-87)	74 (61-89)	0.5
Baseline FEV1/FVC ratio	0.8 (0.8-0.9)	0.8 (0.8-0.9)	0.7
∆FVC (0-12 mo), ml	-90 [(-290)-35]	-280 [(-505)-(-73)]	0.01

Data are median (interquartile intervals, IQI). A. Differential blood parameters assessed at baseline. B. Pulmonary lung function parameters were assessed either at baseline or, in the case of ∆FVC, as a loss between baseline and 12 months post-sampling. Baseline is defined as 0 months and the time of CH assessment. Data were analyzed with Fisher exact tests, using Bonferroni corrections for multiple comparisons (significance level ste at $p<0.05$). Details on statistical tests are described in the Supplementary methods. MCV= mean corpuscular volume; RDW= Red blood cell distribution width, FVC=forced volume capacity, FEV=forced expiratory volume.

Supplementary Table 3. Associations of IPF progression and ΔFVC with CH, adjusted for basic characteristics, comorbidities, antifibrotic therapy and baseline disease severity

**Regression Model**	**Outcomes**					
	**Progression**			**ΔFVC**		
	OR	95% CI	p-value	Estimate	SE	p-value
CH, basic characteristics	3.4	1.3-8.9	0.01	-152.7	71.6	0.03
CH, basic characteristics, comorbidities	4.1	1.5-11.5	0.006	-177.3	70.7	0.01
CH, basic characteristics, antifibrotic therapy and baseline severity	3.9	1.3-12	0.01	-164.5	72.3	0.03

Data are outcomes of logistic (Progression) or linear regression (ΔFVC) assessing their associations with CH. Model 1 includes adjustment for age, sex, smoking history, and family history of pulmonary fibrosis, model 2 includes an additional adjustment for comorbidities including malignancies and coronary heart disease and model 3 includes an additional adjustment for antifibrotic therapy and baseline disease severity. Disease progression was determined 12-months post-sampling, as follows: $\geq$10% absolute FVC loss alone (n=62), $\geq$5% absolute FVC loss and radiologic progression (n=7) or by radiologic progression alone (n=20), as described in Supplementary methods. Data included in the linear regression model do not include patients whose disease progression was based on radiological criteria only.

Supplementary Table 4. Frequency of comorbidities in IPF patients with and without CH mutations

Comorbidity (%)	No CH	CH	*p*-value
GERD	12	15	0.8
PAH	17	9	0.3
Hypertension	36	50	0.2
Coronary heart disease	32	26	0.5
Other cardiac disease	31	17	0.1
Malignant disease	21	11	0.2

Data are percent comorbidities in IPF patients. In addition, COPD frequency was also analyzed using criteria described in the Supplementary Methods. Six non-CH-mutated and one CH-mutated patients were classified as GOLD stage 1 (n=1, non-CH), 2 (n=3 and 1, non-CH and CH, respectively), 3 (n=1, non-CH) or 4 (n=1, non-CH). One patient with CCUS (clonal cytopenia of unknown significance) was part of the CH cohort. Malignant comorbidities included cancers that were treated without recurrence (prostate, basal cell, penis). Data were analyzed using Fisher tests, as described in the Supplementary methods. GERD=gastroesophageal reflux disease, PAH=pulmonary arterial hypertension.
